# Incidence of Acute Myeloid Leukemia after Breast Cancer

**DOI:** 10.4084/MJHID.2011.069

**Published:** 2011-12-22

**Authors:** Caterina Giovanna Valentini, Luana Fianchi, Maria Teresa Voso, Morena Caira, Giuseppe Leone, Livio Pagano

**Affiliations:** Hematology Institute, Catholic University, Rome, Italy

## Abstract

Breast cancer is the most frequent cancer among women and the leading cause of death among middle-aged women. Early detection by mammography screening and improvement of therapeutic options have increased breast cancer survival rates, with the consequence that late side effects of cancer treatment become increasingly important. In particular, patients treated with adjuvant chemotherapy regimens, commonly including alkylating agents and anthracyclines, are at increased risk of developing leukemia, further enhanced by the use of radiotherapy. In the last few years also the use of growth factors seems to increase the risk of secondary leukemia. The purpose of this review is to update epidemiology of therapy-related myeloid neoplasms occurring in breast cancer patients.

## Epidemiology and Mortality Of Breast Cancer

Excluding skin cancers, breast cancer (BC) is the most common malignancy among women in developed country, accounting for about one-third of all new cancer cases in the United States (*n*= 230480; 30%), and it is the second leading cause of cancer death among women. Despite the high incidence, the mortality rate is low (15%), and, as a result of early diagnosis and the increasing use of adjuvant therapy, there is a rising number of long-term survivors.^1^,^2^ Several studies have reported an increased incidence of acute myeloid leukemia (AML) after treatment of BC, with evidence of a dose-intensity relationship. It is estimated that 1 every 20 patients will develop a secondary non-breast cancer after 10 years, which corresponds to a 22% increase of relative risk, particularly for secondary AML and myelodisplastic syndromes (MDS).^3^–^5^

## Differences Between Therapy-Related and Secondary Acute Myeloid Leukemia

Therapy-related acute myeloid leukemia or myelodisplastic syndromes (t-AML/MDS) are collectively known as therapy-related myeloid neoplasms (t-MN), included among “Acute myeloid leukemias and related precursor neoplasms” in the 2008 WHO classification.^6^ The term “therapy-related” leukemia is descriptive and based on patient’s history of exposure to cytotoxic agents. The latency between primary diagnosis and therapy-related disease ranges from few months to several years, with a median of about two years, depending in part on the cumulative dose and/or the dose-intensity of the preceeding cytotoxic therapy, as well on the exposure to specific agents.^7^–^9^ Besides “therapy-related” forms, there are AML/MDS defined as “second malignancy” arising as a second cancer after a previous diagnosis of a neoplasm treated with surgery alone.

Currently accounting for 10–20% of all cases of AML,^10^ the outcome of patients with t-AML compared with that of *de novo* AML, has been historically poor, with a higher frequency of poor-risk cytogenetics and shorter survival times.^11^–^13^ Patients are often poor candidates for intensive AML therapy because of protracted damage from prior cytotoxic therapy and, in some cases, for the persistence of their primary disorder. Moreover, t-AML is relatively resistant to conventional therapies used for *de novo* leukemias.

## Pathogenesis of t-MN After Breast Cancer

Chemotherapy with DNA-targeted antiproliferative drugs in the adjuvant setting has contributed to significant progress in the management of BC, substantially increasing the number of long-term survivors. As the risk of developing cancer increases with age, longer survival is associated with an increased probability of new cancer occurrence, particularly of developing t-AML/MDS. In the majority of cases they are represented by AML, but a secondary acute lymphoblastic leukemia is possible, although less common.^14^,^15^ In the last decades the type of solid tumors preceding t-MN has changed: among 3026 newly diagnosed AML, there were 142 of 200 t-MN with a previous history of solid cancer, with BC representing the most common neoplasm (52%). The median latency between diagnosis of primary malignancy and the occurrence of t-AML was four years, and was shorter in patients younger at the time of primary malignancy diagnosis or treated with anthracyclines and/or topoisomerase-II inhibitors.^10^

Several studies have reported an increased risk for AML in BC patients treated with adjuvant therapy (Table 1 and Table 2), but it remains unclear if t-AML represents a truly stochastic event or if individual susceptibility plays a role.^16^ Already 40 years ago Metcalf et al demonstrated a correlation between acute leukemia and BC and hypothesized common risk factors for both diseases.^17^ This observation has been confirmed in various series of patients showing that there is an increased risk of developing AML in patients with BC treated with surgery alone, or with family history of BC, so that individual susceptibility for development of multiple tumors and a possible association between the two diseases must be hypothesized.^18^–^20^ It is currently difficult to define individual susceptibility, because only few pathological conditions, above all constitutional and genetically determined, are known to predispose to leukemia. The interaction between the genotoxic effects of chemotherapy or ionizing radiation and the “host” is influenced, among others, by genetic polymorphism in drug metabolism and DNA repair processes, which may increase individual susceptibility to these agents. Furthermore, the observation of secondary leukemias in patients who did not receive chemio- or radiotherapy for their primary tumor suggests the existence of a common predisposing condition, possibly a general *cancer* susceptibility.

A population-based study from a French Cancer Registry evaluated the risk of developing a new primary invasive cancer during the first five years of follow up for 14353 cancer patients (breast, colorectal and prostate cancer), comparing with the expected numbers, based on primary cancer incidence rate using the standardized incidence ratio (SIR). Overall, 690 second cancers were registered, including 15 AML. In particular, among 5663 women treated for BC, 10 developed t-AML, which results into a greater risk than the general population (SIR=8.26, *p*<0.05).^21^ AML risk after a prior BC was also examined in an Australian retrospective population-based study and this risk was compared to that of survivors after a prior diagnosis of hematological malignancies and other cancers combined. Among 183123 women diagnosed with BC, 158 (0.09%) subsequently developed AML, with the result that women with a prior diagnosis of BC were 2.6 times more likely to develop AML compared to the general female population (*p*<0.001). Although the incidence of AML rose sharply with age in all cohorts, the age-specific relative risk was highest in the 30–49 age groups and decreased with increasing age. An age-dependent risk of a subsequent diagnosis of AML was confirmed in women <50 years and in the range 50–64 years with previous BC, but not in those older than 65 years, if compared with the expected incidence of AML. A similar age-dependent pattern was observed for second BC and ovarian cancers; this association may be explained by either chemotherapy exposure or an interaction between therapy and genetic predisposition.^22^ On the other hand, Patt et al evaluated the risk of AML in older women treated with modern schedules, demonstrating that while older women treated with adjuvant chemotherapy had more than 50% increased risk of AML, the absolute increase in risk at 10 years was low (1.8% in treated patients *versus* 1.2% among patients who did not received chemotherapy).^23^

## Role of Adjuvant Chemotherapy

Adjuvant chemotherapy for BC has undergone major changes, expanding from node-positive women to lower risk patients. Anthracycline-containing regimens have shown superiority in comparison to cyclophosphamide, methotrexate and 5-fluorouracil (CMF). Incorporation of taxanes (paclitaxel and docetaxel) into anthracyclines-based schedules yielded an additional benefit in both disease-free survival (DFS) and overall survival (OS) in most studies, and dose-dense drug administration have shown to be more effective than the conventional dosing schedule.^24^–^28^ These novel therapeutic strategies have resulted into a considerable improvement of BC survival, but also into an increased t-AML/MDS rate.^29^–^35^ Of note, it must be kept in mind that a under-reported incidence of overall t-MN in the different registries is likely, and difficult to accurately estimate, because of an inadequate coding, not specific for t-AML or t-MDS.

### Alkylating agents

In the past, alkylating agents were the class of antineoplastic drugs unequivocally associated with t-MN (Table 1). The antineoplastic activity of these drugs is related to their ability to damage DNA by methylation or DNA inter-strand crosslinks formation, interfering with normal DNA replication. Alkylating agent-related AML typically develops after an average latency of 5–7 years, and overt leukemia is often (up to 70% of cases) proceeded by a dysplastic phase.^9^,^11^ Fisher et al reported that the 10-year cumulative risk of AML was increased in patients treated with surgery followed by melphalan-based chemotherapy compared to those treated with surgery alone (1.29% *versus* 0.27%, respectively).^30^ In the following years the leukemogenic potential of cyclophosphamide has emerged. Several studies indicated that the risk for developing AML/MDS among patients with early-stage BC treated with adjuvant chemotherapy containing standard dose cyclophosphamide is higher than that of the general population,^31^,^36^ although the risk of developing t-MN in patients treated with melphalan is 10 times higher than that of patients who received cyclophosphamide.^32^ In fact the risk for AML appears negligible in patients treated with CMF regimens, provided that cyclophosphamide is given at standard dose.

### Anthracyclines

The latency period between exposure to anthracyclines and the onset of leukemia is usually about 2 years, and generally there is no previous myelodysplastic phase (Table 2). In order to assess the risk of developing AML and MDS after exposure to epirubicin-based regimen, Praga et al reviewed 7110 patients treated with epirubicin and cyclophosphamide in 19 randomized clinical trials in 2005. At a median follow up of eight years the cumulative probability of AML or MDS was 0.55%; however the risk increased in relation to the cumulative doses of both agents, ranging between 0.37% in patients received standard regimen and 4.97% for those treated with higher doses.^37^ Similar results were obtained with doxorubicin-based regimen. In a large French case-control study, the risk of t-AML/MDS in women treated for BC was higher in those who received mitoxantrone-based chemotherapy than in those given anthracyclines.^38^ Smith et al performed a combined analysis of six adjuvant studies conducted by the National Surgical Adjuvant Breast and Bowel Project group using regimens containing both doxorubicin and cyclophosphamide, and reported a 5-year incidence of AML ranging from 0.3% to 1.2%, with an increased risk for greater dose intensity.^39^ The importance of dose intensity was also confirmed with the “intense dose-dense” regimen epirubicin, paclitaxel and cyclophosphamide every 2 weeks, which proved more effective than standard schedule epirubicin/cyclophosphamide and improved event-free and overall survivals, but was also more toxic with four cases (0.6% of patients) of t-AML/MDS.^26^

Antineoplastic activity of taxanes appears to be related to their ability to promote microtubular assembly and to inhibit microtubular disassembly. A SEER database analysis did not document an increased risk of secondary malignancies with these drugs.^23^ A 7-year follow-up of a trial comparing doxorubicin/cyclophosphamide (AC) *versus* docetaxel/cyclophosphamide (TC) in early BC, reported no secondary leukemia in the TC arm, compared to two cases in 510 patients (0.4%) in the AC arm.^25^

## Role of Granulocyte Colony-Stimulating Factors (G-CSF) and Radiotherapy

Recently, increasing numbers of women receiving adjuvant chemotherapy for BC have also received granulocyte stimulating factors to reduce the myelosuppressive effects of dose-intense chemotherapy. *In vitro* data suggest that G-CSF may increase the risk of AML/MDS, but its leukemogenic effect is still debated. An analysis of the SEER-medicare population-based database including 5510 women with BC treated with adjuvant chemotherapy, found that the addition of G-CSF is associated with a doubling of the risk of subsequent AML or MDS when compared with chemotherapy alone, even if the absolute risk is low.^40^ In the analysis of six trials described by Smith et al and mentioned above, the incidence of therapy-related leukemia was sharply elevated in patients treated with intensified regimens that required G-CSF support (relative risk 6.16, *p*=0.0001).^39^ On the other hand, Patt et al did not find an increased risk for AML in elderly (>65 years) BC patients, who received G-CSF during the first years after diagnosis as part of adjuvant therapy.^23^ Similarly, in the Cancer and Leukemia Group B 9741 phase III trial, patients received dose-dense regimens plus filgrastim support, but had no increased risk of developing AML or MDS compared to those treated with the same regimen at conventional schedule without G-CSF.^27^ Finally, a systematic review of 25 randomized clinical trials was recently conducted to evaluate the risk of AML or MDS in patients receiving chemotherapy for solid malignancies and lymphomas with or without the addition of G-CSF. At a median follow up of 54 months, the estimated relative risk for AML/MDS with G-CSF-supported chemotherapy was 1.92, with an estimate absolute increase in risk of 0.4%;^41^ however, although this increased risk, these data cannot distinguish between the potential causal effects as a result of the growth factor and of dose-intensified systemic chemotherapy, so that the potential toxicities of G-CSF needs further study.

Radiotherapy may also play a significant role (Table 3). A cohort study analyzing clinical records of BC patients with the aim of evaluating the long-term effect of radiotherapy on the risk of second cancers reported a total of 387 malignancies (7.3%) in 5248 women, with eight patients developing leukemia (0.15%), seven in the group treated with radiotherapy, *versus* one case only in the group not receiving radiotherapy. The relative risk adjusted for chemotherapy and hormone treatment was 6.67 (95% CI 0.76–58.00) and the median time from exposure was 4.5 years, with the suggestion of a raised incidence of leukemia within the first two or more years after radiotherapy.^42^ Similarly, in a previous study, the risk of developing AML resulted four-fold increased with the use of radiotherapy (HR 4, 95% CI 1.4–11.8) and by seven folds when radiotherapy was combined to chemotherapy (HR 7.2, 95% CI 1.4–36.3).^33^

## Summary and Final Remarks

- In BC survivors, there is a small but significant and increasing number of secondary myeloid neoplasms after adjuvant chemotherapy, particularly after treatment with alkylating agents and/or topoisomerase II inhibitors, that lead to two distinctly and different forms of t-MN.
- The risk of leukemia appears very low if the cumulative dose of anthracyclines and cyclophosphamide is not very high. Clinical trials attempting to improve therapeutic benefit by dose escalation need to take into account the increased risk for leukemia, when assessing potential benefits and risks.
- The incidence of t-MN appears to be also increased in patients treated for BC with surgery alone, and these cases are not “therapy-related”. Thus, t-MN may be part of a cancer-risk syndrome involving BC, and possibly a general *cancer* susceptibility.
- A raised risk of t-MN is associated with radiotherapy, particularly for women treated after the menopause.
- The concurrent use of G-CSFs as supportive care in order to deliver intensive adjuvant chemotherapy could further enhance this risk, so that their use should be limited to the settings with available strong evidence.

## Figures and Tables

**Table 1:** Risk of therapy-related myeloid neoplasms in breast cancer patients receiving adjuvant chemotherapy with alkylating agents.

Reference	Breast cancer patients (*n*)	Therapy	t-MN (*n*)	Cumulative risk (%)
Valagussa et al, 1994 [36]	2465	CMF	3	0.23 (at 15 y)
Bernard-Marty et al, 2002 [34]	255	CMF	0	0 (at 5 y)
Praga et al, 2005 [37]	1427	CMF	1	0.07 (at 8 y)
Ejlertsen et al, 2007 [24]	629	CMF	2	nr
Hershman et al, 2007 [40]	3330	Cyclophosphamide-based regimens	40	1.20

CMF: cyclophosphamide, methotrexate, and fluorouracil; nr: not reported.

**Table 2:** Risk of therapy-related myeloid neoplasms in breast cancer patients receiving adjuvant chemotherapy with anthracyclines.

Reference	Breast cancer patients (*n*)	Therapy	t-MN (*n*)	Cumulative risk (%)

Bernard-Marty et al, 2002 [34]	267	Epirubicin-based regimens	3	0.9 (at 5 y)

Smith et al, 2003 [39]	6018	Doxorubicin + CTX	21	0.12 (at 8 y)
	2545	Doxorubicin + CTX + G-CSF	22	0.86 (at 8 y)

Praga et al, 2005 [37]	7110	Epirubicin-based regimens	28	0.55 (at 8 y)

Campone et al, 2005 [35]	3653	Epirubicin-based regimens	8	0.34 (at 9 y)

Ejlertsen B et al, 2007 [24]	584	CEF	1	nr

Hershman et al, 2007 [40]	1569	Doxorubicin-based regimens	18	1.14

Patt et al, 2007 [23]	5213	Anthracycline-based regimens	nr	1.53 (at 10 y)

Burnell et al, 2010 [28]	2104	Antracycline-based regimens	8	nr

CTX: cyclophosphamide; CEF: cyclophosphamide, epirubicin and fluorouracil; nr: not reported.

**Table 3:** Risk of therapy-related myeloid neoplasms in breast cancer patients receiving radiotherapy.

Reference	Breast cancer patients (*n*)	Therapy	t-MN (*n*)	Cumulative risk (%)

Renella et al, 2006 [33]	2292	Radiotherapy alone	7	1.9
	1119	Radiotherapy + chemotherapy	2	1.7

Howard et al, 2007 [4]	99275	Radiotherapy alone	221	0.22 (at 10 y)

Hershman et al, 2007 [40]	2837	Radiotherapy alone	38	1.33

Schaapveld et al, 2008 [5]	31000	Radiotherapy alone	13	1.28 (at 10 y)

Martin et al, 2009 [3]	420076	Radiotherapy +/− chemotherapy	450	0.91 (at 5 y) for range 15–49 years; 1.14 (at 5 y) for range 50–64 years; 1.76 (at 5 y) for >65 years

Zhang et al, 2011 [41]	1779	Radiotherapy alone	7	6.67 (at 4.5 y)
	553	Radio- + hormonal therapy		
	293	Radio- + chemotherapy		
	25	Radio- + hormonal+chemotherapy		
	2650	Total		
